# Prediction Tools for Unfavourable Outcomes in *Clostridium difficile* Infection: A Systematic Review

**DOI:** 10.1371/journal.pone.0030258

**Published:** 2012-01-24

**Authors:** Claire Nour Abou Chakra, Jacques Pepin, Louis Valiquette

**Affiliations:** Department of Microbiology and Infectious Diseases, University of Sherbrooke, Quebec, Canada; Charité, Campus Benjamin Franklin, Germany

## Abstract

**Context:**

Identifying patients at risk for adverse outcomes of *Clostridium difficile* infection (CDI), including recurrence and death, will become increasingly important as novel therapies emerge, which are more effective than traditional approaches but very expensive. Clinical prediction rules (CPRs) can improve the accuracy of medical decision-making. Several CPRs have been developed for CDI, but none has gained a widespread acceptance.

**Methods:**

We systematically reviewed studies describing the derivation or validation of CPRs for unfavourable outcomes of CDI, in medical databases (Medline, Embase, PubMed, Web of Science and Cochrane) and abstracts of conferences.

**Results:**

Of 2945 titles and abstracts screened, 13 studies on the derivation of a CPR were identified: two on recurrences, five on complications (including mortality), five on mortality alone and one on response to treatment. Two studies on the validation of different severity indices were also retrieved. Most CPRs were developed as secondary analyses using cohorts assembled for other purposes. CPRs presented several methodological limitations that could explain their limited use in clinical practice. Except for leukocytosis, albumin and age, there was much heterogeneity in the variables used, and most studies were limited by small sample sizes. Eight models used a retrospective design. Only four studies reported the incidence of the outcome of interest, even if this is essential to evaluate the potential usefulness of a model in other populations. Only five studies performed multivariate analyses to adjust for confounders.

**Conclusions:**

The lack of weighing variables, of validation, calibration and measures of reproducibility, the weak validities and performances when assessed, and the absence of sensitivity analyses, all led to suboptimal quality and debatable utility of those CPRs. Evidence-based tools developed through appropriate prospective cohorts would be more valuable for clinicians than empirically-developed CPRs.

## Introduction

In the decade that followed the emergence of the *Clostridium difficile* hypervirulent strain NAP1/BI/027 in North America and Western Europe, health professionals have been increasingly challenged by the burden of this infection, its frequent recurrences, severe complications and deaths [1]–[4].

Currently, the management of severe, complicated *Clostridium difficile* infection (CDI) is based on high-dose vancomycin, with or without intravenous metronidazole, intensive care unit (ICU) admission, vasopressor support and colectomy for a few selected patients [5], [6]. Most patients present initially with similar symptoms, and identifying which ones might progress to these dreadful complications is difficult.

After a long period of stagnation, novel therapeutic approaches are being developed for CDI. Fidaxomicin, recently licensed by the Food and Drug Administration, is more effective than vancomycin in avoiding recurrences [7], [8]. Monoclonal antibodies were also proven to be effective in preventing recurrences, in a phase 2 trial [9]. Both fidaxomicin and monoclonal antibodies are unfortunately very expensive. Thus, it will become increasingly important to identify, early in the course of the disease, which patients would be most likely to benefit from these novel therapies, from closer follow-up, or both [10], ultimately to decrease CDI-related morbidity and mortality.

Clinical prediction rules (CPRs), which can improve the accuracy of medical decision-making, could address some of the aforementioned challenges in CDI management, and facilitate the conduct of clinical trials evaluating experimental therapeutic approaches. Several CPRs for CDI complications have been proposed over the years, but none has gained widespread clinical acceptance. We therefore performed a systematic review of all publications that aimed to derive or validate a CPR to predict recurrences, complications and mortality in patients diagnosed with CDI.

## Methods

### Study selection

A systematic review was performed according to PRISMA guidelines [11] (**checklist S1**) using an electronic search (**Text S1**) of all studies published since January 1978 (the year that *C. difficile* was identified as the etiological agent of pseudomembranous colitis [12], [13]), in English, French or Spanish. The search was limited to humans and used the following online libraries and databases: Medline, PubMed, Cochrane, Embase and Web of Science. Furthermore, we reviewed abstracts submitted to conferences organised by the American Society for Microbiology, the Society for Healthcare Epidemiology of America, the Infectious Diseases Society of America, the Association of Medical Microbiology and Infectious Disease Canada, the Anaerobe Society of the Americas and the European Society of Clinical Microbiology and Infectious Diseases. In addition, the reference lists of identified CPRs were searched manually (cross-referencing). The final electronic search was performed on 30 October 2011.

### Inclusion criteria

Publications from all sources were gathered in one file and duplicates were removed. A first screening of titles and abstracts followed by a full-text review were performed by CAC in order to identify studies that: i) focused on *C. difficile* as the main pathogen; ii) measured at least one relevant outcome: severity of the infection, complications, mortality, treatment failures or recurrences; and iii) developed or validated a model or risk score, a prediction rule, an index or a scale. Quality control on 10% of electronic search results was performed (LV) for the first screening of abstracts, as well as for all included studies. Reviewers had a good agreement concerning eligible studies (87%). Disagreements were resolved by a third party (JP).

### Data collection

The following data were extracted by two reviewers (CAC and LV), from each included publication, into a standardized matrix: definitions of main outcomes, description of the study design, study population, sample size, statistical analyses and main results in relation with the objectives of the review. Authors were directly contacted in case of missing or incomplete data.

### Quality assessment

The quality of CPR derivation studies in full-text publications was assessed qualitatively through a description of biases and limitations, and quantitatively through the attribution of points for the derivation and validation methodologies. The criteria of Laupacis [14], McGinn [15], and May [16] were used as standards for the essential steps in the derivation, validation and reporting of CPRs. A total of 20 points could be reached for the derivation methodology and of 10 for the validation, with one point assigned to each step (**Text S2**). The impacts of the CPRs (potential effects if implemented into practice) and the subsequent work to determine their accuracy were considered optional in the publications on the derivation of a CPR and were not included in the quality assessment.

## Results

### Search results

**Figure 1:**
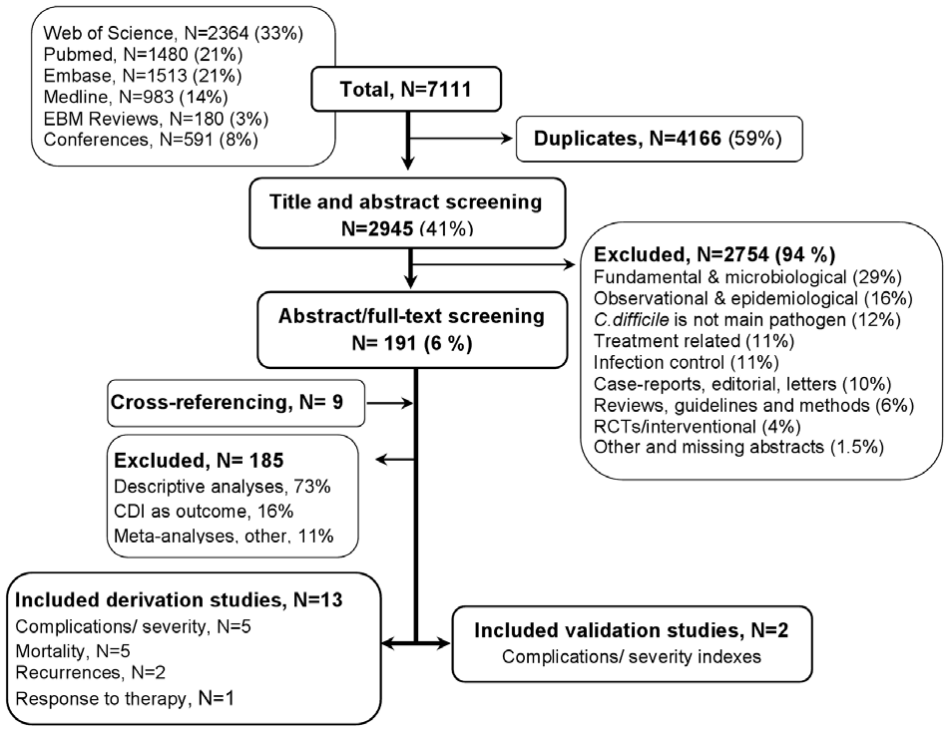
Flow chart of publications' inclusion and exclusion.

The electronic search led to 7111 publications. After excluding duplicates, 2945 (41%) were reviewed by title and abstract (**Figure 1**). According to pre-defined criteria, 2754 (94%) publications were excluded. Following the full-text review, we retained 15 studies: 13 studies on the derivation of prediction rules or models, including or not a validation process, and two studies on validation alone. Overall, we identified two derivation studies on recurrences, five on complications/severity including mortality, five on mortality alone and one on response to treatment. The two validation studies focused on severity indices.

### I. Derivation studies

**Figure 2:**
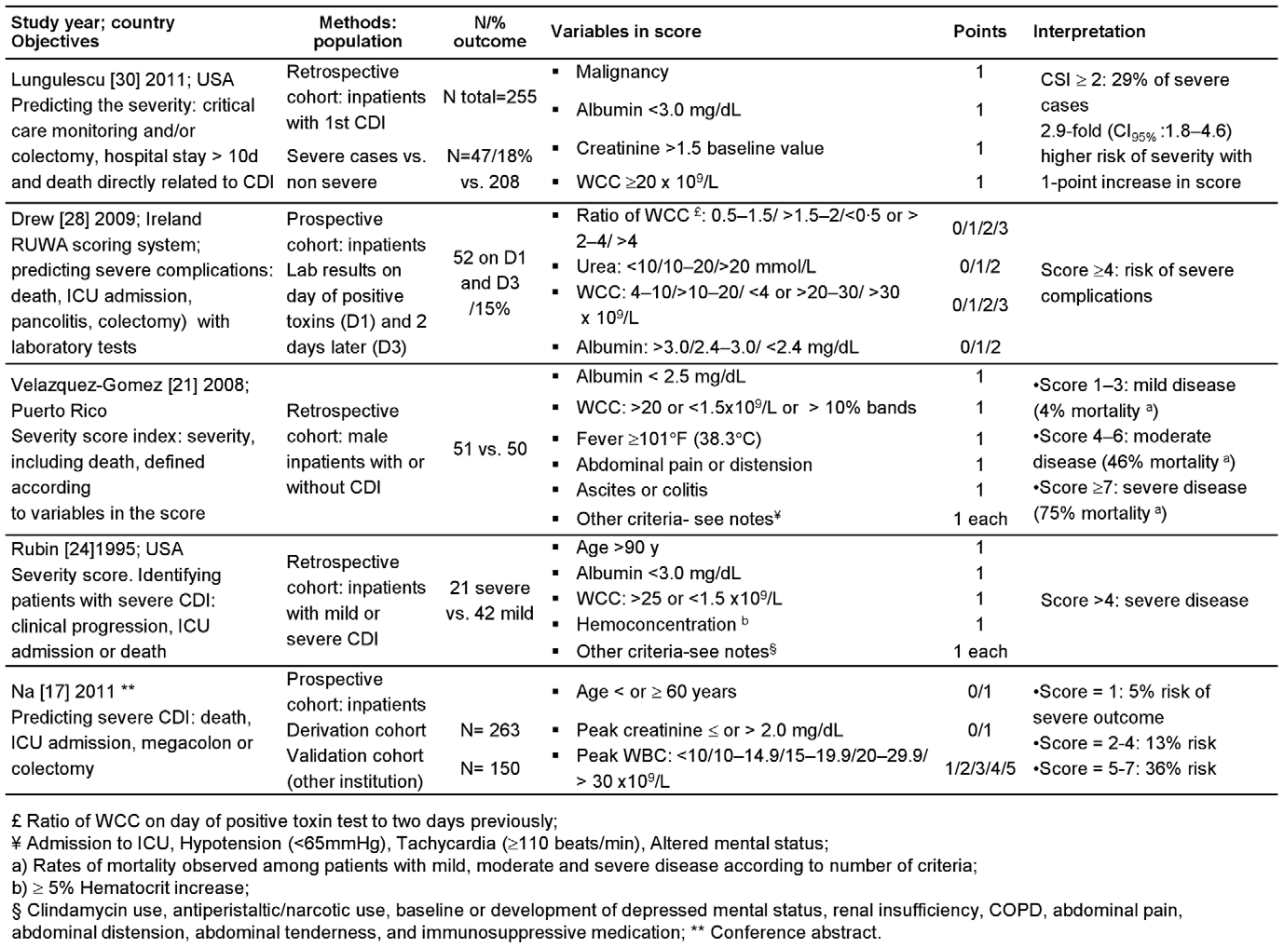
Prediction scores for complications of CDI.

**Figure 3:**
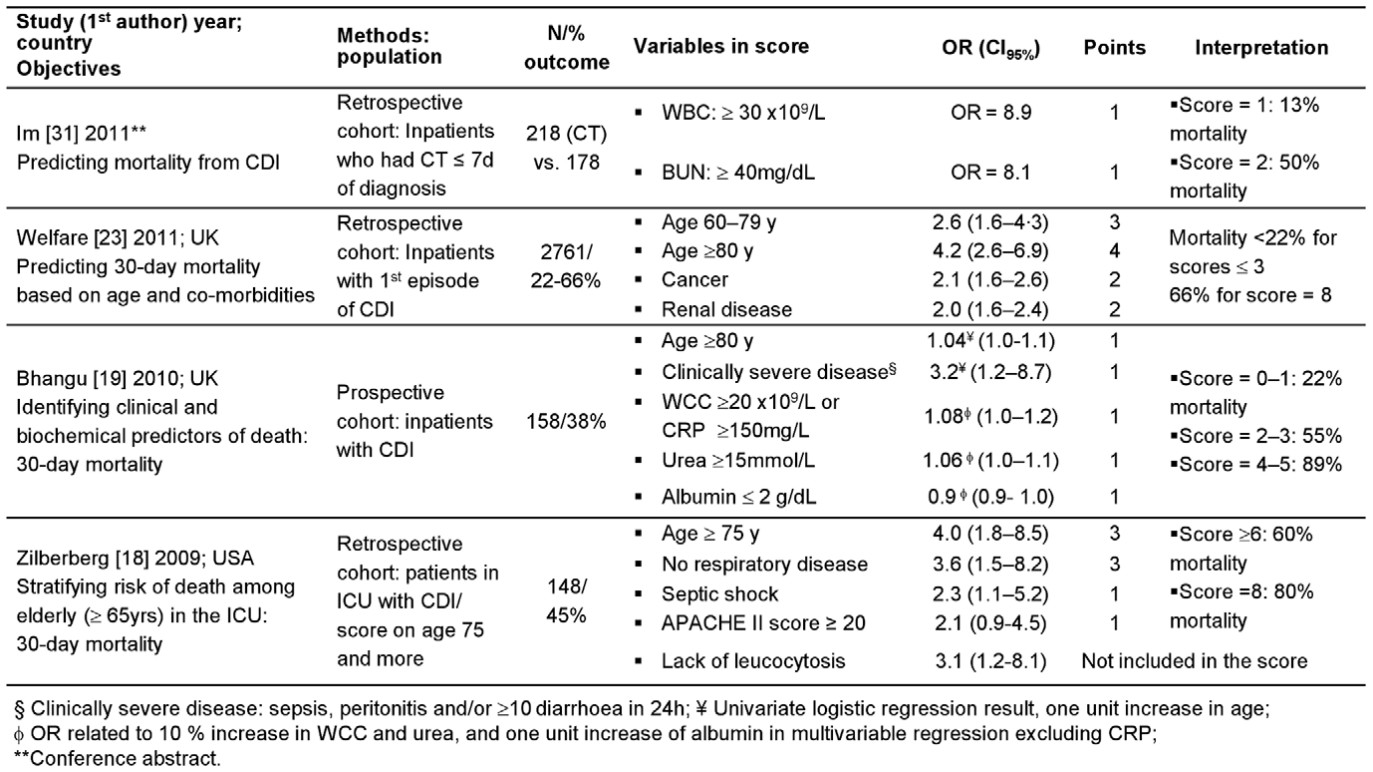
Prediction scores of mortality related to CDI.

**Figure 4:**
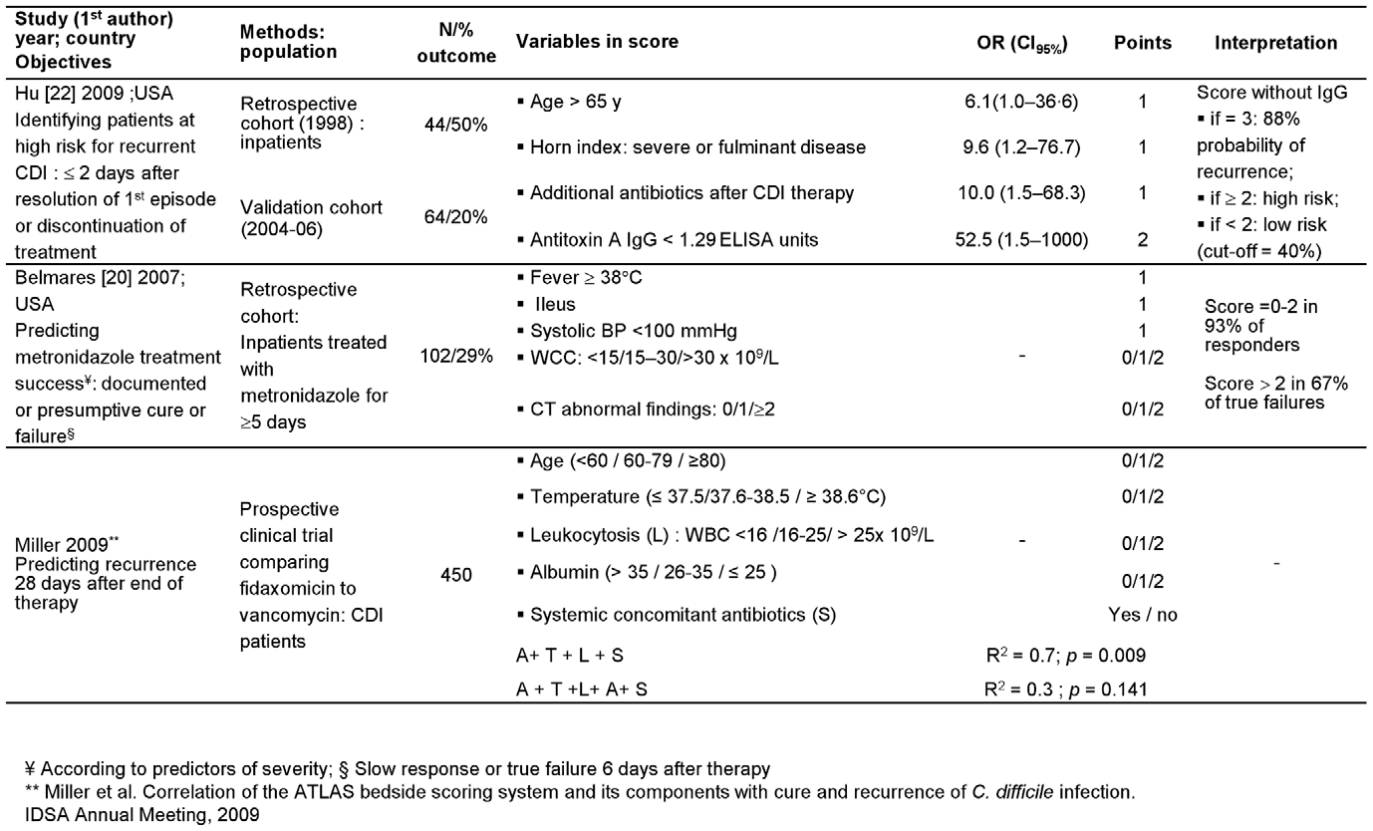
Prediction scores of recurrence of CDI and treatment success.

As shown in Figures 2–4, leukocytosis (white cell count, [WCC]) was included in the majority of the scores (n = 9) and hypo-albuminemia in half of them (n = 6). In studies with univariate analyses, prediction models were based on long lists of criteria (between 4 and 13). Few criteria remained significant after multivariate analyses, with older age being the most frequent (n = 5). All but one [17] studies on complications included only univariate associations. In addition, we included Miller's study (Correlation of the ATLAS bedside scoring system and its components with cure and recurrence of *C. difficile* infection. IDSA Annual Meeting, 2009) on predicting recurrence 28 days after end of therapy (Figure 4). The score correlated with cure much better than with recurrence (R^2^ = 0.85 vs. 0.32), and correlated with recurrence only among patients receiving fidaxomicin (R^2^ = 0.7 vs. 0.02 for those given vancomycin). This score was used to predict mortality in a second cohort (n = 308; mortality = 8%) by comparing the median score in survivors and non-survivors: the difference was significant (*p* = 0.0002). (Chopra et al. ATLAS-A bedside scoring system predicting mortality due to *C. difficile* infection in elderly hospitalized patients. IDSA Annual Meeting, 2010).

### Study data and quality assessment

Overall, only four studies reported the incidence of the outcome of interest [18]–[21], eight used a retrospective design for derivation and six used multivariate analysis. Prospective collection of outcomes was performed in only one study [19] and blinding to assess outcomes in two studies [18], [19]. Assigning points to each variable in the scores proportionally to the variables' coefficients was performed in only four studies [17], [18], [22], [23]. Therefore, only four studies obtained more than 10/20 points with regard to the quality of derivation methodology (Table 1).

**Table 1:** Assessment of quality of CPRs in the derivation process.

	Study (1^st^ author)								
	Lungulesco [30]	Hu [22]	Zilberberg [18]	Bhangu [19]	Belmares [20]	Welfare [23]	Drew [28]	Velazquez-Gomez [21]	Rubin [24]
**A. Clearly defined outcomes**	√	√	√	√	√	√	√	-	√
**B. Prospective predictors**	-	-	-	√	-	-	-	-	-
**C. Description of subjects**									
Inclusion criteria	√	√	√	√	√	√	-	√	√
Method of selection	√	√	√	√	√	√	-	√	√
Demographic characteristics	√	√	√	√	√	-	-	√	√
Clinical characteristics	√	√				-			
**D. Sample size** a	√	-	√	√	-	√	-	-	-
**E. Comparison group**	√	-	√	√	√	-	-	√	-
**F. Univariate analysis** b	√	-	√	√	-	-	-	√	√
**G. Multivariate analysis**	-	√	√	√	-	√	-	-	-
**H. Accuracy**									
Sensitivity	√	√	-	-	√	-	√	-	-
Specificity	√	√	-	-	√	-	√	-	-
PPV	√	√	-	-	-	-	√	-	-
NPV	√	√	-	-	-	-	√	-	-
Likelihood ratios	-	-	-	-	-	-	-	-	-
AUC	√	√	√	-	√	-	-	-	-
Confidence intervals	-	√	√	-	√	-	√	-	-
**I. Blinding in assessing outcomes**	-	-	√	√	-	-	-	-	-
**J. Scores proportional to β** c	-	√	√	√	-	√	-	-	-
**Total quality score**	13	13	12	11	9	6	6	5	5

a Sample size: at least 10 outcomes per predictor variable;

b Univariate analysis of predictors;

c β coefficient: estimate in multivariate logistic regression.

Furthermore, only two studies used a validation cohort [17], [22]. A total of 8/10 points was assigned to Hu's [22] and of two to Zilberberg's studies [18]. Seven studies reported validation and performance parameters of scores or models (Table 2). To our knowledge, Rubin [24] did not validate their scoring system, but it was later validated by Fujitani [25]. Welfare [23] assessed the internal validity of their score through a Chi-square comparison between the two halves of a split derivation cohort. [21], [26].

**Table 2:** Reported validation and performance parameters of prediction scores or models (95% confidence interval).

Study	Model	Sensitivity	Specificity	PPV§	NPV¢	AUC£	Diagnostic accuracy
***Derivation step***							
**Lungulescu** [30] **: Cut-off score: 2 of 4 criteria**	History of malignancy + WBC ≥20×10^9^/L + albumin <3·0 mg/dL + creatinine >1·5× baseline	82%	65%	38%	93%	0.8	69%
**Drew** [28] **: Cut-off score ≥4**	Lab results on day1 (Ration WCC, WCC, urea and albumin)	80% (39–96)	77% (74–79)	25% (12–30)	98% (93–100)	-	-
	Lab results on day3	63% (32–86)	82% (79–85)	29% (15–40)	95% (91–98)	-	-
	Lab results on day 1+ day 3	100% (59–100)	70% (66–70)	-	-	-	-
**Im** [31] **: 2-variables model**	WBC ≥30×10^9^/L + BUN ≥40 mg/dL	-	-	-	-	0.9	-
	Low risk (score = 0) vs. high (score ≥1)	100%	62%	-	-	-	-
	Model + moderate and severe pericolonic stranding	100%	82%	-	-	0.9	-
**Belmares** [20]	Optimal score = 2.5	67%	93%	-	-	0.9 (0.8–1.0)	-
***Validation step***							
**Belmares score in Fujitani** [25]	Variables in the score against CDC definition of severity	74%	93%	70%	97%	-	-
**Hu** [22]	Age + Horn's index + additional antibiotics	54% (25–81)	77% (63–87)	37% (16–62)	87% (73–95)	0·8 (0·7–0·9)	72% (59–82)
	Age + Horn's index + additional antibiotics + IgG	38% (9–76)	83% (59–96)	50% (12–88)	75% (51–91)	0·6 (0·4–0·8)	69% (48–86)
**Zilberberg** [18] **; Cross-validation: bootstrap; 10% of sample; 25 iterations**	Age ≥75 y + septic shock + no respiratory disease + Apache II score ≥20	-	-	-	-	0·7 (0·7-0·8)	-
**Rubin** [24] **in Fujitani** [25]	Variables in the score against CDC definition of severity	63%	87%	36%	95%	-	-

§ PPV: positive predictive value;

¢ NPV: negative predictive value;

£ AUC: area under the ROC curve.

Other limitations were identified. Hu [22] used risk factors that had been associated with recurrence in a previous study [27]. Velazquez-Gomez [21], Drew [28] and Belmares [20] empirically derived a scoring system using laboratory data and factors previously associated with severe disease, refractoriness to treatment and mortality. The severity of disease in the study of Velazquez-Gomez [21] was defined *a priori* according to the presence of risk factors, and therefore mortality was high (75%) in patients fulfilling more than seven criteria, including hypotension, tachycardia and ICU admission. Older age was one of the variables in Zilberberg's score [18] initially derived to predict mortality among the elderly (Figure 3), but the weight given to age was potentially over-estimated by being also included within the APACHE II score [29]. The ARC score (age, renal disease and cancer) [23] was initially based on age and co-morbidities, but. ORs were rounded down and significative variables with OR between 1 and 1.5 were left out. Miller used participants in a clinical trial to develop their prediction score (Figure 4). Clinical trials are carried out with restrictive inclusion criteria, which somewhat limits the external validity of this score if used in the general population of patients with CDI.

### Performance measures

Frequencies of observed or predicted outcomes of interest by the CPRs were low across studies, ranging between 15% and 66%. In Na's study [17], the maximum possible score (n = 7) was equivalent to only 36% of the risk of severity, including death. In Lungulescu's study [30], 29% of severe cases had a score ≥2 among four possible criteria. On the other hand, with only two clinical parameters (WCC and BUN), Im [31] predicted 50% of the risk of mortality, and with >2 among 7 criteria Belmares [20] predicted only 67% of treatment failures.

When reported, sensitivities (38%–82%), specificities (62%–93%), positive predictive values (PPV; 25%–50%) and diagnostic accuracy (69%–72%) were relatively low (Table 2). The area-under-the-curve (AUC) values were modest; the highest (0.9; IC_95%_ = 0.8–1.0) corresponded to a score of 2.5 over 7 in Belmares' cross-validation [20] although few patients experienced true treatment failures (Figure 4). None of the included studies reported analysis of likelihood ratios, sensitivity analyses, the potential effects if the CPRs were implemented into practice, nor a follow-up to determine accuracy in real-life use.

### II. Validation studies

The score of Velazquez-Gomez [21] (severity score index) was prospectively validated by Toro [26]. A cohort of CDI patients (male veterans; n = 54) with a score corresponding to mild, moderate and severe disease at diagnosis (Figure 2) was followed for 90 days to assess the severity and mortality. The validity of the score was assessed through Chi-square comparisons. Need of ICU care and mortality correlated with high severity in the index (*p*<0.05 and *p* = 0.005 respectively). In quality assessment, this study was assigned 2 points over 10.

Fujitani [25] analysed eight severity score indices, most of them with no published data concerning their derivation and validation. They were rather validated in a prospective cohort (n = 184) using the Center for Disease Control and Prevention definition of severity which includes the presence of at least one of: admission to ICU, surgery for toxic megacolon, bowel perforation, refractory colitis, or 30- day death attributed to CDI [6]. Indices had moderate sensitivities (63–84%), low PPV (19–57%), and poor concordance with CDC definitions (Kappa score: 0.18 to 0.69). Apart from the scores of Rubin [24] and Belmares [20] included in our review, the other indices were mainly used for definition of CDI severity at diagnosis and were not derived for prediction [32]–[35]. None of those indices assessed a risk of an unfavourable outcome.

## Discussion

To our knowledge, this is the first systematic review of prediction tools for unfavourable outcomes of CDI, offering to practitioners a comprehensive summary and assessment of available CPRs. Standard methodology for systematic reviews was followed with rigorous quality control. Numerous key words and medical databases were used, and a very large number of publications were scanned in order to retrieve all available CPRs of interest. Furthermore, in order to identify CPRs in grey literature: conference abstracts of six major infectious diseases societies were searched.

Most CPRs on unfavourable outcomes of CDI were developed as secondary analyses using cohorts assembled for other purposes. CPRs included in the current systematic review presented several methodological limitations that could explain their very limited use in clinical practice. Except for WCC, albumin and age, there was much heterogeneity in the variables used in various scores, and most studies were limited by small sample sizes. Eight of the included models used a retrospective design, and one used the population of a clinical trial. It is generally suggested that predictive variables should be collected prospectively, and therefore more accurately, in a process established specifically for the development and the validation of clinical rules [14], [16]. Only four studies reported the incidence of the outcome of interest, even if this information is essential to evaluate the potential usefulness of a given model in populations other than the one used for its derivation [36]. In addition, analysis of likelihood ratios in the validation process is independent from the incidence of the outcome [37] but none of the included studies reported any such measures. Multivariate analyses are also recommended for the derivation process in order to account for the confounding and interaction between variables [14]. Only five studies performed multivariate analyses, but their results need to be interpreted cautiously since the confidence intervals for the adjusted odds ratios were wide.

The majority of CPRs were developed to predict the likelihood of complications or severe CDI, including death. Only two CPR were published on recurrences, one from a small retrospective cohort and the other from a clinical trial with a restricted population. Recurrence is an important problem associated with CDI, causing significant morbidity [38], [39]. The availability of costly new treatments potentially lowering the rate of recurrence [7], [9] increases the importance of identifying at the time of diagnosis patients at high risk of recurrence. None of the current recurrence scores seems to be able to predict recurrence with accuracy. The development and validation of recurrence scores should be a priority.

We designed a scale to assess the quality of methodology through objective criteria. The derivation process of included studies was rather weak, the two best ones fulfilling a maximum of 13 criteria over 20. The lack of weighting variables, of validation, calibration and measures of reproducibility, the weak validities and performances when assessed, and the absence of sensitivity analyses all led to suboptimal quality and very debatable utility of those clinical rules or prediction models for health practitioners [40].

Other severity scores or indices are available in the literature but since no data were available on their derivation process, they were not included in this study: some of them were validated in Fujitani [25] using the CDC definition of severity [6]. These indices had moderate sensitivities, low predictive values, and poor concordance with the CDC definition (0.18 to 0.69). Moreover, included indices measured variables and risk factors at different time points after CDI diagnosis [25].

Our systematic review has some limitations. As there were relatively few prediction tools (only 13 identified), inclusion criteria had to be permissive and we also examined publications with limited information: four abstracts, a letter [28] and studies that used only univariate analyses. Conference abstracts are not always available online for reviewers.

### Conclusion

In conclusion, available prediction tools for unfavourable outcomes of CDI present many methodological biases and weak validities, limiting their usefulness in clinical settings. Evidence-based tools developed through appropriate prospective cohorts would be more valuable for clinicians than empirically-selected clinical factors.

## Supporting Information

Text S1 Electronic search
(DOCX)

Text S2 Quality assessment criteria for derivation and validation steps.
(DOCX)

Checklist S1 PRISMA criteria.
(DOC)
