# Supplementary material for: Prediction Tools for Unfavourable Outcomes in Clostridium difficile Infection: A Systematic Review
Source: PLoS One. 2012 Jan 24;7(1):e30258. doi: 10.1371/journal.pone.0030258 (PMC3265469; doi:10.1371/journal.pone.0030258)
Supplement: Text S1 — Electronic search (DOCX) [file pone.0030258.s001.docx]

**Text SI- Electronic search**

***Databases used for the electronic search***

Medline: Ovid Medline®, In-Process & Other non-indexed citations since 1950 and PubMed;

Cochrane Library**:** EBM Reviews - Cochrane Central Register of Controlled Trials 4th Quarter 2010, EBM Reviews - Cochrane Database of Systematic Reviews 2005 to December 2010; Embase: 1980-2011

Web of Sciences: SCI-EXPANDED 1979-2011

***Keywords***

“Clostridium difficile/ Clostridium difficile-associated diarrhea/ Clostridium difficile-associated disease AND (diarrhea OR diarrhoea OR colitis OR pseudomembranous OR enterocolitis OR enteritis OR antibiotic-associated disease) AND (sensitivity OR specificity OR predict$ OR index OR score OR model OR factor OR grad$ OR decision rule OR decision technique OR prognosis OR risk index OR risk score OR risk model OR risk scale”.

***Conferences ‘abstracts***

American Society for Microbiology: ICAAC; 2008, 2009 and 2010;

The Society for Healthcare Epidemiology of America (SHEA): 2007-2008

The Infectious Diseases Society of America (IDSA): 2001, 2003-2007 and 2009

The Association of Medical Microbiology and Infectious Disease Canada (AMMI): 2006-2010; The Anaerobe Society of the Americas: 2008;

The European Society of Clinical Microbiology and Infectious Diseases (ESCMID): 2000-2010.
