# Supplementary material for: Prediction Tools for Unfavourable Outcomes in Clostridium difficile Infection: A Systematic Review
Source: PLoS One. 2012 Jan 24;7(1):e30258. doi: 10.1371/journal.pone.0030258 (PMC3265469; doi:10.1371/journal.pone.0030258)
Supplement: Text S2 — Quality assessment criteria for derivation and validation steps. (DOCX) [file pone.0030258.s002.docx]

**Text S2 – Quality assessment criteria for derivation and validation steps**

***Criteria for derivation***

1. Clearly defined outcomes: a clear and precise definition of the outcome is reported;
2. Prospective predictors: predictors are assessed prospectively;
3. Description of subjects clearly reported:

- Inclusion criteria
- Method of selection
- Demographic characteristics
- Clinical characteristics

1. Sample size: at least 10 outcomes per predictor variable;
2. Adequate comparison group: non-significant differences in univariate analysis for relevant characteristics;
3. Univariate analysis of predictors;
4. Multivariate analysis of predictors;
5. Accuracy; reported:

- Sensitivity
- Specificity
- Positive predictive value
- Negative predictive value
- Likelihood ratios
- ROC curve and area under the curve
- Relative confidence intervals

1. Blinding in assessing outcomes;
2. Assigned scores are proportional to β-coefficients estimating the effect of predictors in the multivariate analysis.

***Criteria for validation***

1. Prospective validation in a different cohort;
2. Criterion standard applied to all patients in derivation and validation cohort to determine true outcome;

C. Accuracy:

- Sensitivity
- Specificity
- Positive predictive value
- Negative predictive value
- Likelihood ratios
- ROC curve and area under the curve
- Relative confidence intervals

D. Sample size: at least 10 outcomes per predictor variable.
